# Supplementary material for: Chiral optical response of planar and symmetric nanotrimers enabled by heteromaterial selection
Source: Nat Commun. 2016 Oct 13;7:13117. doi: 10.1038/ncomms13117 (PMC5065623; doi:10.1038/ncomms13117)
Supplement: Supplementary Information — Supplementary Figures 1-2, Supplementary Note 1 and Supplementary References. [file ncomms13117-s1.pdf]

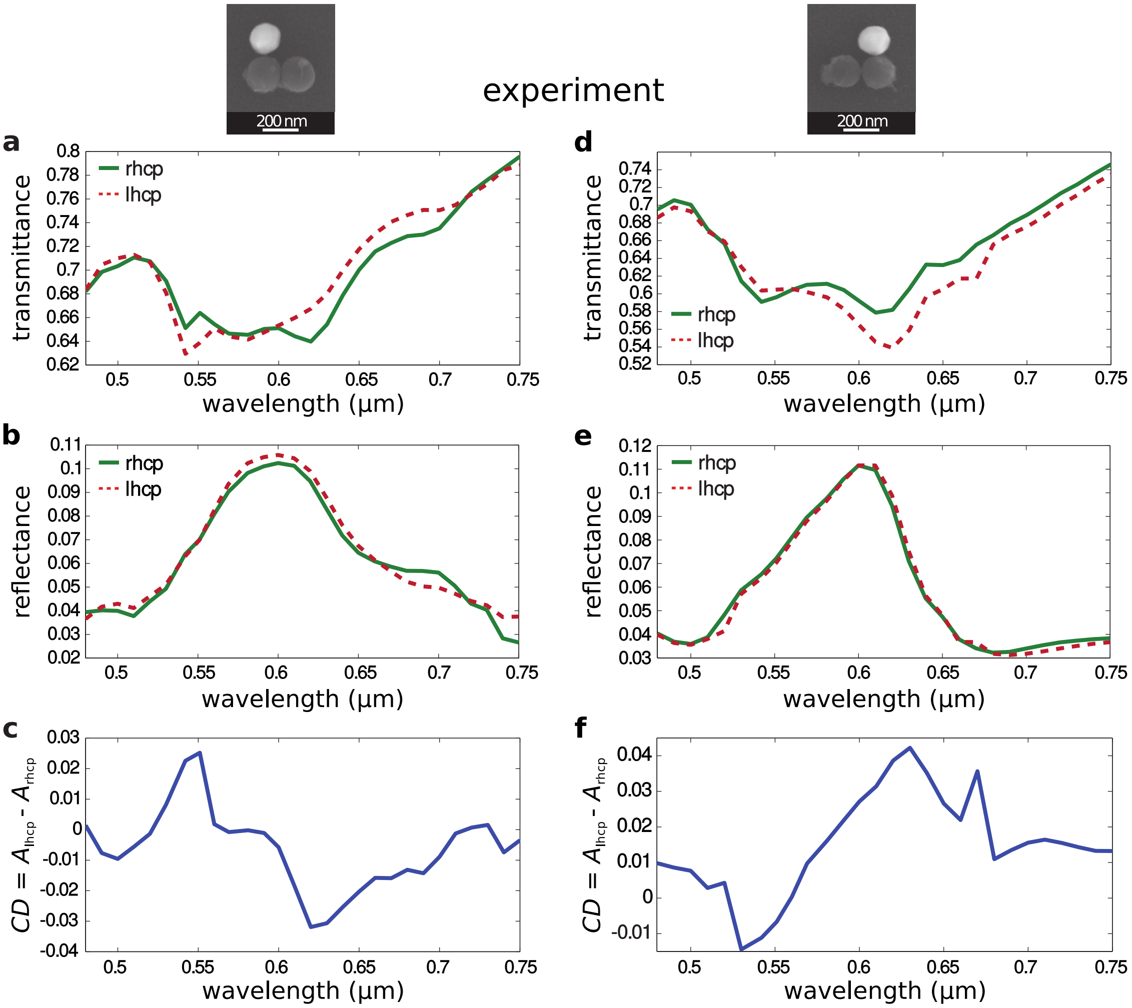

Supplementary Figure 1 – **Comparison of the optical response of heterogeneous nanosphere trimers of opposite handedness.** (a)-(c) Experimental transmittance, reflectance and CD spectra for the trimer shown in the main text. For dimensions please see main text. d)-(f) Experimental results for a trimer of opposite handedness but slightly different geometrical parameters in comparison to the trimer discussed in the main text. The particle sizes as retrieved from the SEM micrograph are approximately 164 nm for the Au particle (top right), 171 nm for the lower left Si particle and 173 nm for the lower right Si particle. The gap between neighboring particles is close to zero.

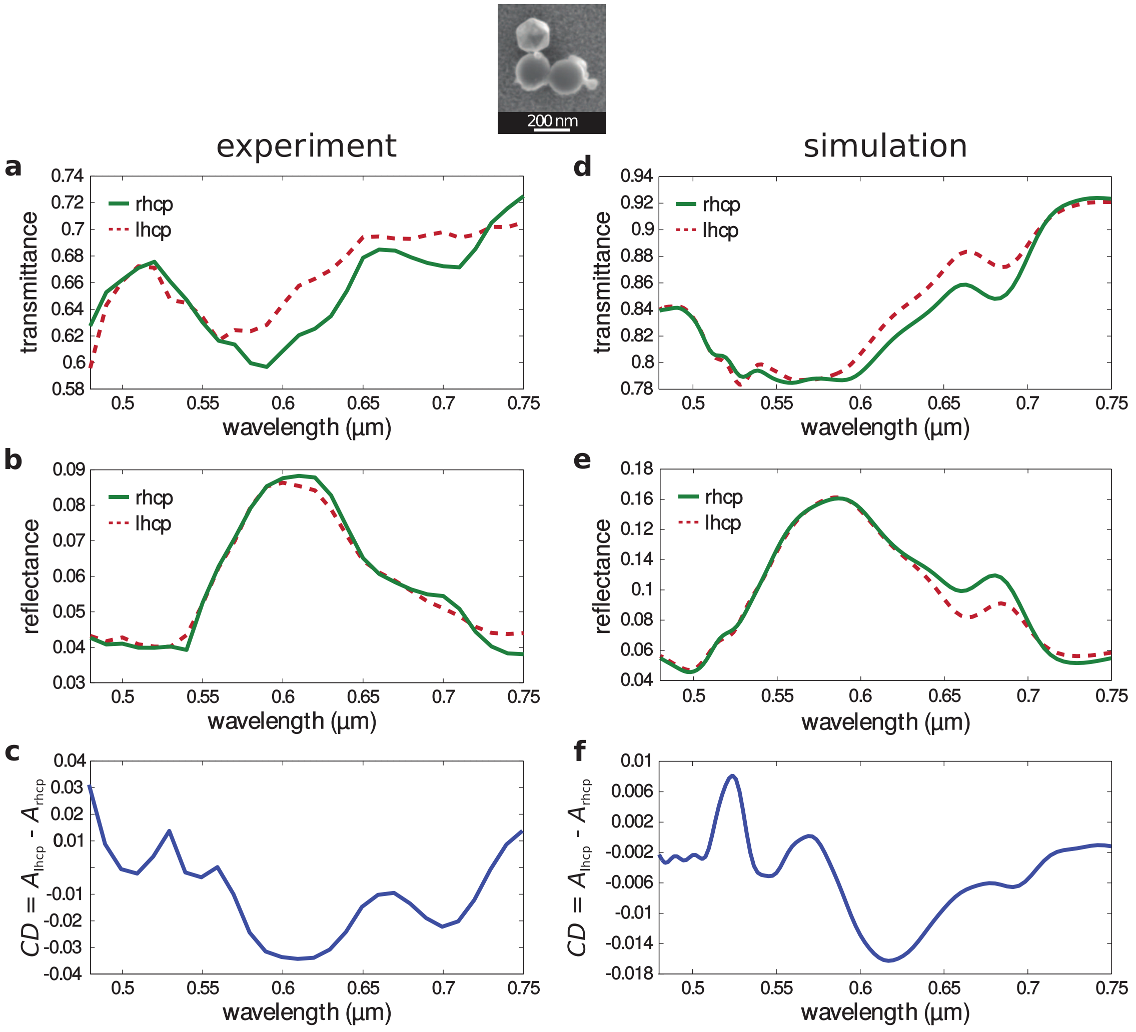

Supplementary Figure 2 – **Optical response of a heterogeneous nanosphere trimer (same handedness as structure shown in main manuscript.** (a)-(c) Experimental transmittance, reflectance and CD spectra for another trimer of the same handedness but slightly different geometrical parameters in comparison to the trimer discussed in the main text. (d)-(f) Corresponding numerically calculated spectra taking into account the actual sizes and particle shapes retrieved from the SEM micrograph. Particle sizes have been retrieved from the SEM micrograph and used in the shown simulations. The particle diameters are approximately 182 nm for the Au particle (top left), 178/201 nm for the lower left, slightly elongated Si particle and 191 nm for the lower right Si particle. The gap between neighboring particles is zero.

SUPPLEMENTARY NOTE 1 – Additional Experimental Results

To further prove the proposed concept of heterometerial selection for the creation of optically chiral nanostructures, we performed additional experiments on trimers of the same and mirrored geometry with respect to the one shown in the main manuscript. The experiments were performed as described in the Methods section (see also Ref. 1 for more details about the setup). The corresponding datasets for a trimer of opposite handedness are plotted in supplementary Fig. 1 (d - f) in comparison to the data for the trimer already discussed in the main text (see Fig. 1(a - c)). As expected, the observed curves for lhcp and rhcp excitation change places and the CD spectra are flipped.

In Fig. 2, we also show additional experimental and numerical results for a trimer exhibiting the same handedness but slightly different geometrical parameters (see Fig. caption for details) in comparison to the structure discussed in the main text. In the simulations, the actual particle diameters, shapes and positions as indicated in the figure caption were taken into account. As can be seen, a small change in the diameters or shapes of the involved (Si) particles can cause visible changes in the spectra (compare supplementary Figs. 1 and 2 (a-c) with Fig. 5 in the main text). This allows the fine-tuning of the resonant response of heterogeneous trimers.

Supplementary References

1. Banzer, P., Peschel, U., Quabis, S. & Leuchs, G. On the experimental investigation of the electric and magnetic response of a single nano-structure. *Opt. Express* **18**, 10905–10923 (2010).
